# The Meaning of Sedentary Behavior for People with Spinal Cord Injury: A Reflexive Thematic Analysis

**DOI:** 10.3390/healthcare14183068

**Published:** 2026-09-17

**Authors:** Nathan T. Adams, Sarah V. C. Lawrason, Diane Rakiecki, Rachael Brears, Kathleen A. Martin Ginis

**Affiliations:** 1 School of Health and Exercise Sciences, University of British Columbia, Kelowna, BC V1V 1V7, Canada; 2 Centre of Chronic Disease Prevention and Management, University of British Columbia, Kelowna, BC V1V 1V7, Canada; 3 International Collaboration on Repair Discoveries, University of British Columbia, Vancouver, BC V5Z 1N1, Canada

**Keywords:** people with disabilities, prolonged sitting, constructivism, qualitative research, wheelchair users

## Abstract

**Highlights:**

We interviewed people with spinal cord injuries to explore their perspectives on sedentary behavior. Participants were familiar with the term sedentary behavior and understood how it applied to their daily lives. Because many people with spinal cord injuries use wheelchairs, they are especially attuned to the experience of sitting and how it affects them. For individuals who feel they sit frequently and have limited control over their sitting time, media messages such as “sitting is the new smoking” can feel inappropriate or stigmatizing.

**What are the main findings?**

- People with spinal cord injury know about sitting and the symptoms it exacerbates for them. Their experience with sitting is centered around the health challenges felt by spinal cord injury.
- Media messaging related to sedentary behavior is exclusive of people with spinal cord injury.

**What are the implications of the main findings?**

- Sedentary behavior is an important area of study for people with spinal cord injury, but research efforts must be tailored to the specific needs and priorities of this population.
- Sedentary behavior media should be more inclusive.

**Abstract:**

**Background/Objectives**: On average, people with spinal cord injury (SCI) accumulate 10–13 h of sedentary behavior a day. It is unclear what people with SCI know about sedentary behavior and its potential impact. The purpose of this study was to examine how people with SCI interpret sedentary behavior, particularly sitting, and media messaging about sedentary behavior and sitting. **Methods**: We used semi-structured interviews to explore the meaning of sedentary behavior among people with SCI. We recruited 12 participants who were medically stable, living in the community, had a chronic SCI, and primarily used a wheelchair when outside of the home (M age = 49.2 years old, SD = 12.7; M = 17.3 years since injury, SD = 8.6). We analyzed the data through reflexive thematic analysis. **Results**: The five themes generated were as follows: (i) understanding of sedentary behavior is constructed through personal experiences and social interactions; (ii) sedentary time exacerbates symptoms and symptoms increase sedentary time; (iii) activity is intentional, while interruptions of sedentary behavior are incidental; (iv) life as a sitter: sedentary behavior becomes part of the identity; (v) “that’s not for me”: media messages exclude people with SCI. **Conclusions**: People with SCI have a general understanding of sedentary behavior and its negative effects. Their experience with sedentary behavior is centered around their symptoms, which are often unique to SCI. Sitting is perceived as unavoidable, but also as restorative and necessary. Media messages related to sedentary behavior are ableist and exclude individuals who use wheelchairs.

## 1. Introduction

Sedentary behavior has garnered growing scientific and public interest over the past two decades [1,2], owing to its established associations with increased risk of cardiovascular disease, depression, and all-cause mortality [3,4,5]. However, limited research has examined sedentary behavior among individuals living with chronic spinal cord injury (SCI) [6], a population characterized as being at the lowest end of the physical activity (PA) participation spectrum [7,8]. People with SCI are underrepresented in sedentary behavior research and there is a lack of understanding as to how people with SCI think about and experience their sedentary time. Before further inquiry into the characteristics, antecedents and consequences of sedentary behavior in people with SCI can be meaningfully advanced, it is essential to center the voices and lived experiences of this community. Accordingly, the present study aims to explore the knowledge and experience of sedentary behavior among individuals with SCI.

Globally, more than 15 million individuals are living with SCI [9]. Individuals with SCI typically experience high volumes of sedentary behavior, averaging 10–13 h per day which is at least four hours more than estimates for the general population [10,11,12]. Increased sedentary behavior is related to an increased risk of non-communicable diseases and reduced subjective well-being in the general population. For people with SCI who already experience an elevated baseline risk of cardiovascular and metabolic diseases, these effects may be further amplified [13,14]. Moreover, sedentary behavior is linked to secondary complications specific to SCI, including pressure injuries and neurogenic bladder dysfunction [15,16,17]. These effects of sedentary behavior remain untested in SCI and are instead hypothesized based on evidence from other populations and related physiological pathways. Importantly, the sedentary experiences of people with SCI may be fundamentally different from people without disabilities. This may be a result of the unique physiological challenges and symptoms that are present after SCI, as well as the fact that many lack viable postural alternatives to sitting, reclining, or lying down. Consequently, conventional approaches to defining, conceptualizing and even talking about sedentary behavior may not be entirely appropriate for this population. In this paper, we draw on principles of the social model of disability to situate our work and language choices away from an individual or medicalized framing, recognizing disability is shaped by social, cultural, and environmental barriers [18], while noting that this model did not serve as the primary analytic framework for our thematic analysis. Our understanding is built on the current best practices for inclusive language and framing in Canada and the input from the author who has lived experience of spinal cord injury.

There is a lack of phenomenological research exploring the experiences of people with SCI during sedentary behavior. Insights from similar research on PA can help shape expectations for this study. Prior work shows that people with SCI perceive exercise as beneficial for their physical health, mental well-being, independence, and social participation [19]. However, their experiences with PA and sport can be shaped by factors such as pre-injury experiences, their ability to fully participate, and how sport aligns with their life trajectory [20,21]. When people with spinal cord injuries are enabled to fully engage in PA and exercise, they can experience meaningful positive emotions and they feel it is beneficial for their overall health [22]. If sedentary behavior were treated as the direct opposite of PA, one might expect people with SCI to view it as harmful to their physical health, mental health, independence, and social engagement. Yet these behaviors are not opposites [23], and the context surrounding sedentary time—such as who individuals are with and what they are doing—can strongly shape how sedentary behavior is experienced. This underscores the need for research that explores the unique and nuanced experiences of sedentary behavior among people with SCI.

Despite increasing media and public attention to sedentary behavior which has increased public awareness, most adults remain unfamiliar with its formal definitions and associated health consequences [24]. Media messaging may shape awareness, but its influence on behavior—particularly among people with SCI—remains largely untested [25]. Even within scientific communities, the term “sedentary” has been inconsistently and sometimes improperly applied [23]. The Sedentary Behavior Research Network defined sedentary behavior as “any waking behavior characterized by an energy expenditure ≤1.5 metabolic equivalents, while in a sitting, reclining or lying posture” [26]. While this terminology was originally developed for use in scientific contexts, mainstream discourse and media frequently reduce sedentary behavior to the concept of “sitting”, which oversimplifies its complexity. The meanings people with SCI attach to sedentary behaviors, including sitting, warrant closer examination.

Public discourse on sedentary behavior is typically negative. Media portrayals of sedentary behavior often adopt ableist narratives, contributing to exclusionary discourse around physical inactivity [27]. Sensationalized headlines such as “Too Much Sitting Is Killing You” and “Sitting Is the New Smoking” [28,29] hyperbolize the effects of sitting [30] to a level that risks alienating and stigmatizing individuals with disabilities, including those with SCI. There is evidence that fear appeal messaging is ineffective for changing behaviors, particularly when perceived efficacy for changing the behavior is low [31]. Such messaging may inadvertently discourage people with SCI from engaging with public health information about sedentary behavior, further widening gaps in awareness and understanding relative to the general population. However, little is known about how individuals with SCI think about sedentary behavior or how they interact with media discourse surrounding sitting and sedentary time.

The purpose of this study was to explore how people with SCI think about and experience sedentary behavior, and their responses to media messages about these behaviors. To address these issues, we developed a qualitative study based on three research questions: (i) What do people with SCI know about sedentary behavior? (ii) How do people with SCI experience sedentary behavior? (iii) What do people with SCI think about the media portrayal of sedentary behavior?

## 2. Methodology

### 2.1. Qualitative Approach

This study was guided by a constructivist paradigm which emphasizes understanding individuals’ subjective experiences and perceptions [32]. Because each participant’s perspective is shaped by their unique context, variation across accounts is not only expected but valued as an essential source of insight within these scientific methods. This approach stands in contrast to positivist methodologies, which seek to identify a singular, generalizable experience across participants and extend this experience as truth across all people within the population. Although participants may at times draw on more positivist or constructionist understandings of their experiences, the design of this study, including the methods, interview, and analysis, was explicitly grounded in constructivism. Therefore, the knowledge generated within this study is grounded in a relativist ontology and shaped through a subjectivist epistemology, wherein researcher–participant interactions actively co-created the data and findings [33].

To ensure methodological coherence between our ontological and epistemological assumptions [34], we conducted semi-structured interviews and analyzed the data through reflexive thematic analysis [35]. The flexible nature of the interview guide enabled the researcher to tailor each interview to the ideas that participants deemed meaningful, rather than rigidly adhering to predefined questions. Reflexive thematic analysis, as conceptualized by Braun and Clarke [36,37], further supported this flexible and responsive approach. Such alignment with constructivist principles reinforces our central goal: to deeply understand the meanings individuals construct from their lived experiences.

To support meaningful insight into the experiences of individuals with SCI, the research team engaged in reflexive practices throughout data generation and analysis. As the lead author and interviewer, I (NA) maintained a reflexive journal to document evolving thoughts and decisions during both the interview and analytic phases. In addition, collaboration with critical friends (SL, KMG) and people with lived experience with SCI (DR) enriched the process: they contributed to the development of the interview guide, provided input on coding and theme development, and co-authored the final report. In addition, this research question and study concept were informed by engagement with community. The research question and methods were designed following Integrated Knowledge Translation Guiding Principles [38]. In this study, this meant collaborating with partners with whom we had established long-term relationships, ensuring that individuals with lived experience were meaningfully involved in decision-making, and providing partners with sufficient training to support their active engagement throughout the project. These practices improved the acceptability of the project overall and reduced researcher bias. We were intentional in selecting methods aligned with our epistemological and ontological positions to ensure the findings were appropriately contextualized.

### 2.2. Recruitment

This study received ethical approval from the Behavioral Research Ethics Board at The University of British Columbia [H24-01509]. Participants were recruited through social media posts on our lab account and accounts of SCI partnership organizations. Local and provincial SCI organizations also assisted with outreach by distributing recruitment materials via their websites and mailing lists. In addition, we posted study advertisements at a rehabilitation hospital and at local adapted gyms to broaden community outreach. The resulting sample included a mix of genders, injury levels, and injury completeness. Although not designed to be statistically representative, this diversity supported the generation of rich, situated accounts consistent with the aims of reflexive thematic analysis.

Eligible participants met the following inclusion criteria: (i) living with SCI for a minimum of one year, (ii) 18 years of age or older, (iii) able to read and converse in English, (iv) residing in Canada during the time of this study, and (v) primarily using a wheelchair for mobility outside the home. After completing the eligibility screening, participants were provided with a digital consent form. Upon participation, each individual received a USD 50 Amazon gift card as compensation for their time. Braun and Clarke critique proceduralist approaches for determining sample size that rely on saturation, arguing instead for reflexive decision-making [39]. We determined that our sample was complete in situ, based on the richness and complexity of the data and our growing ability to construct a coherent, compelling analytic story that addressed our research question. In preparation for these decisions, we expected that 10–20 participants would be necessary to collect data rich enough for a proper reflexive thematic analysis.

### 2.3. Data Collection

To support the semi-structured interview approach, an interview guide was developed encompassing three key domains: participants’ knowledge of sedentary behavior, contextual factors influencing their sedentary behavior, and their engagement with and interpretation of media related to sedentary behavior (Supplementary Material File S1). A research team member with lived experience of SCI contributed meaningfully to the guide’s development, ensuring relevance and sensitivity to the target population. All interviews were conducted by the same member of the research team (NA). In keeping with a semi-structured interview approach, interviews followed the topics outlined in the guide, but the interviewer retained flexibility to vary the order of questions and introduce probes as needed. Prior to data collection, the interview guide was piloted twice: once with an individual without SCI and once with an individual with SCI. Feedback from both pilot interviews informed refinements to the guide, enhancing its clarity, responsiveness, and appropriateness for use in this study.

To support the interview process, a reference document was created and shared with each participant during their interview (Supplementary Material File S2) [26,40,41,42,43,44,45]. This document included the scientific definition of sedentary behavior, as outlined by Tremblay et al. [26]. This document was not sent to participants ahead of time. The reference document was not introduced at the same time in each interview; rather, the document was introduced when the topics within were introduced at the discretion of the interviewer. For example, the single slide with the definitions was shared on the Zoom screen for the participant to view if this topic was being discussed. Participants did not click through the reference document at their own pace, and in some cases not all of the slides were shown during a given interview. Definitions of active and passive sitting were also included and introduced when relevant to the conversation along with a definition of activities of daily living [46] and the full PA and sedentary behavior guidelines from the World Health Organization [40]. Additionally, five representative media headlines related to sedentary behavior were shared to elicit reflections on public messaging, including the frequently cited “Sitting Is the New Smoking” [41,42,43,44,45]. These materials were intentionally used to shape discussion around specific messages.

All interviews were conducted via Zoom videoconferencing software (Zoom Workplace, Version: 6.4.12). Videoconferencing was chosen for two reasons. First, this facilitated recruitment across Canada and made participation easier for participants who face transportation barriers. Second, videoconferencing allowed the participants to take part in the interviews from a location of their choosing. This was often their home, which allowed us to collect real-time reflection on sedentary behavior while participants engaged in their day-to-day routines.

Transcripts were automatically generated from the Zoom recording and reviewed for accuracy against the audio recordings before being imported into NVivo (Version 14.23.4) for analysis.

### 2.4. Data Analysis Procedures

Data analysis followed Braun and Clarke’s six-phase reflexive thematic analysis approach [37,47]. Prior to coding, the first author undertook a process of familiarization by reading and listening to each interview multiple times, both to confirm accuracy with the transcript and to engage deeply with the content. Once immersed in the data, coding was conducted inductively using semantic codes to capture the explicit content of participants’ responses. These codes were later collapsed based on meaning, which led to the development of the final themes.

Codes served as foundational tools for generating themes. Theme development was guided by the interpretive synthesis of codes and an ongoing effort to create overarching meaning from the data. We intentionally allowed themes to vary in abstraction so that both interpretive patterns and participants’ direct perspectives were appropriately represented. Multiple iterations of theme generation were undertaken, supported by combining related codes, physically sorting printed code segments into conceptual groupings, and engaging in reflexive journaling to track interpretive decisions. This process was further enriched by collaboration with a critical friend (KMG), who posed probing questions and challenged the boundaries and coherence of the themes, thereby enhancing analytic rigor. Additionally, journaling after interviews, examining overlapping codes, generating themes, and engaging in discussions among co-authors all contributed to strengthening reflexivity. Rather than “validating” codes or themes in a positivist sense, co-authors provided alternative interpretations, questioned assumptions, and supported deeper engagement with the data, consistent with reflexive thematic analysis. This paper was developed following guidelines for reporting qualitative research [48]. To provide context for the quotations in this report, each quote is followed by participant information listed in the following order: gender, neurological level of injury, and completeness of injury. We selected quotations based on their clarity and analytic richness, while ensuring representation across participants to reflect the breadth of experiences in the dataset.

## 3. Results and Analysis

### 3.1. Participants

A total of 68 participants completed the online recruitment questionnaire, and 12 participants were screened, consented, and interviewed before the research team made the in situ decision that the data were rich enough to conclude the interviews. Demographic characteristics of the 12 participants are presented in Table 1. The average length of interview was 56.3 min (SD = 10.1 min).

### 3.2. Themes

Five themes were identified through the process of reflexive thematic analysis.

#### 3.2.1. Understanding of Sedentary Behavior Is Constructed Through Personal Experiences and Social Interactions

Individuals with SCI reported having a general awareness of the concept of sedentary behavior and its detrimental health impacts. When participants were first asked if they recognized the term “sedentary behavior”, most had a vague understanding, as one participant shared:

I don’t recognize it necessarily as a term that has a specific meaning, but I know what both those two words mean. So, I would say sedentary is sitting around, not moving a lot. And behavior is just that, your behavior of sitting. That’s how I would define it.

(P1MTC; Participant pseudonym legend: P# (participant number) M/W (man or woman) T/C/L (thoracic or cervical or lumbar) C/I (complete or incomplete).)

Knowledge of the impacts of sedentary behavior was shaped through participants’ personal experiences, interactions within their social networks, and guidance from healthcare professionals. Participants noted that their insights often stemmed from encounters in medical settings or exchanges with peers. As one participant explained in describing their learning process, “the [impact of sedentary behavior is] learned because I was in the healthcare industry… So it is learned, but then it’s also experience too” (P7WCI).

Beyond externally acquired information, experiential learning played a vital role in shaping participants’ understanding of sedentary behavior. When discussing their knowledge of sitting, many described its direct influence on health outcomes, particularly negative health outcomes. One participant said: “well, it’s not good for your heart, it’s not good for your weight, circulation” (P12WCI).

#### 3.2.2. Sedentary Time Exacerbates Symptoms and Symptoms Increase Sedentary Time

Symptoms significantly influenced the duration of sedentary behavior. Participants often adjusted their posture or increased their physical movement to manage symptoms or to prevent potential health complications related to cardiovascular function, respiratory function, bowel and bladder control, and digestion among other physiological phenomena. Among various experiences, three primary symptoms most consistently prompted changes in sitting time: pain, pressure sores, and fatigue. The symptoms could both induce sedentary time or be the consequence of too much sedentary time. One participant expressed their experience with pain and fatigue by saying:

The less I do, the worse I feel. It’s kind of a catch 22. If I go crazy hard with intervals or races, there were races a couple weeks ago. It’ll take me a few days to recover from that. But yeah, in general, let’s try to keep going.

(P1MTC)

Too little sedentary time could lead to overexertion, provoking pain or fatigue, whereas prolonged periods of sitting could result in discomfort or lethargy. A second participant repeated the “catch 22” phrase from above to describe their experience with pain saying: “maybe I shouldn’t sit so still at times. But then, on the other hand, it’s a catch 22 situation. You’re damned if you do damned if you don’t. Well, you [have] got to let something heal right?” (P2MLI).

One participant reflected on their approach to symptom management, describing a careful balance between maintaining sufficient activity and keeping spasticity under control. Although they valued the brief periods when they were able to walk, extended time out of the chair often triggered bouts of spasticity. They explained, “I’m striking that balance between, you know, being a bit more sedentary versus getting up and moving. So that keeps the spasticity a little bit less” (P9MCI). While participants felt the negative effects of prolonged sitting, they also recognized its restorative role and necessity for overall well-being. One accepted this equilibrium, stating, “It makes more sense to be sedentary kind of throughout the day” (P9MCI). Considerable attention was devoted to identifying a sustainable approach to sedentary behavior that could help reduce unwanted symptoms. These perspectives reframed sedentary behavior as a necessary activity that exists in a balance rather than a purely negative aspect of their routine.

#### 3.2.3. Activity Is Intentional, Interruptions of Sedentary Behavior Are Incidental

Participants were asked whether they deliberately planned interruptions to their sedentary behavior. Most did not consider such intentional interruptions, instead believing that general movement and PA throughout the day were sufficient to counteract the negative effects of sitting.

Some participants were unaware of how much time they spend sitting and did not have reflective moments to trigger planned interruptions. One participant reflected that “it wouldn’t be something I would think about and go [exercise]” (P12WCI). Even when prompted by external cues, such as technology-based reminders, participants often did not act on them. As the previous participant noted, “my Apple watch tells me when to roll, but I ignore it a lot” (P12WCI).

This does not imply that interruptions to sedentary behavior never occurred. Some participants described engaging in spontaneous movement, driven more by habit or an instinctive need to shift position than by conscious planning. One participant illustrated this as follows:

If I’m sitting completely still, staring at a screen or on my phone for 20 min or a half hour? yeah, I need to move and I don’t even think about it. I’m always moving. I don’t even think I sit for 20 min. I mean, I get to a point where I need to move, I mean, unless I’m driving where I’m stuck. But if I’m in my chair, you know the brakes are on for 10 min, and then the brakes come off and I’m twisting, turning anything to get my trunk kind of rotating, arms moving, something right?

(P8MCI)

The previous quote also revealed that some felt an instinctual need to move, which would result in unintentional (with specific regard to sedentary behavior) interruptions and increased light PA. One participant, who previously indicated a lack of focus on reducing sedentary behavior, expressed that they exerted significant energy while completing activities of daily living. They stated, “I do know that my life takes more energy. Doing daily everyday things like going to the bathroom takes me more energy than it does anybody else” (P5WCI), showing how the increased effort required for basic tasks tempered their concerns about sitting and inactivity.

#### 3.2.4. Life as a Sitter: Sedentary Behavior Becomes Part of the Identity

Sitting was deeply ingrained in daily life for participants. This routine was so central that, upon hearing the definition of sedentary behavior, one participant responded, “That’s me” (P4WCC). The act of sitting as experienced through the use of wheelchairs strongly influenced participants’ sense of self. One participant reflected on their decision to use a wheelchair in community settings rather than walk, emphasizing their perceived identity in social interactions.

By being in a wheelchair, I can be more authentic with people because I’m less looking out for my own bodily functions that have been aggravated by the injury. I know that by being in a wheelchair, being able to quickly wheel to the bathroom, or whatever I know, then I can actually sit there and focus on people, and really be my authentic self. Whereas I’ve noticed when I’m walking a bit more, as much as that’s great. It also some ways makes me less authentic, because I’m also worried about taking care of myself physically.

(P9MCI)

The necessity of sitting meant that sedentary behavior became deeply tied to identity, sometimes in strongly negative ways. One participant powerfully likened their inability to walk to a feeling of incarceration. “It’s almost like a prisoner not being able to get out of prison and go into the community and engage with people and socialize because they’re in prison” (P11MTC). Some participants even had these negative identities placed on them by others, like one who said, “My mother used to say, ‘Oh, that’s my wheelchair daughter’” (P12WCI).

While some participants expressed negative feelings about how sitting shaped their identity, others who were very active had broader, more empowered self-definitions. These individuals more strongly self-identified as active persons or athletes than as people with disabilities. One para-athlete, who engaged in rigorous training, described this perspective clearly: “I know that I’m active, like, I’m an athlete” (P6WLI). This identity was echoed among recreational athletes as well. In contrast, less active participants were more likely to define themselves as sedentary and associated their lifestyle with physical inactivity.

The intentionality surrounding sitting became deeply embedded in participants’ routines and how they interacted with their own physical spaces. As one noted: “I’ve strategically planned the places around my home with whatever it is that I need to sit comfortably” (P1MTC). Participants carefully considered the chairs they used, the type of wheelchair, and the duration of sitting. Posture was also adapted based on social context. For example, many chose to sit “properly” around visitors while allowing themselves more relaxed or reclined positions when alone or among close family.

Social identity and professional roles were also influenced by sitting, especially for those who acquired a SCI during their working years. One participant recalled how their vocational options narrowed after injury: “When I broke my back, and then they’re like they’re trying to figure out like what type of careers I could do. It’s like, Oh, well, you’re gonna need a career that’s sedentary” (P10MTC). This led to career suggestions and vocational rehabilitation primarily focused on desk-based roles.

#### 3.2.5. “That’s Not for Me”: Media Messages Exclude People with SCI

When presented with media headlines about sitting, participants tended to distance themselves from inflammatory messaging. Many were familiar with these headlines. While reactions varied, the overarching sentiment was one of skepticism or disconnection. A few participants, however, felt that the headlines convinced them that they should be more active. One physically active participant noted that such headlines prompted him to increase activity levels, stating, “it really concerns me and like I said, I just try and move as much as I can, but at the end of the day I’m not, you know, I’m not standing” (P8MCI). Another participant acknowledged agreement with the general message but dismissed the tone, saying, “it’s a bit blown out of proportion” (P9MCI). Even among those who accepted the factual basis of the headlines, there was a sense of limitation; participants expressed feeling powerless to change their sitting habits but identified increasing PA as a more feasible and motivating alternative.

Most participants expressed that the inflammatory language was exclusive of people with SCI. Most participants said that they did not read the articles that followed these headlines. For example, one said:

When I see this, it’s like it just doesn’t apply to me is how I feel. You asked me how I felt in general. And in general, it’s like whatever. I know the intent of this is to keep people moving so that they are healthier. And I think that’s great. I think if I think about it in depth, it makes me upset that people who can move don’t. Because I would be thrilled if I could go for a walk or a run. And then if I could take it in a step further, it’s like, what? What the fuck? Like I’m probably more fit than 95% of the people that can walk and so but I’m sitting all the time. So this just doesn’t apply to me. That’s how I see it.

(P1MTC)

Participants felt that it was important to discuss sitting. They felt that the messages about sitting are important, and some even shared media articles about sitting with their able-bodied family members. However, most saw the messages as excluding people with SCI and so they simply did not engage with these articles. This was not always the case, though, with one participant voicing their neutrality towards the headlines sharing that they “just ignore it, because it really has no effect on [them]… [the definition] doesn’t really seem like a negative definition” (P10MTC). These divergent accounts were treated as meaningful counter-narratives that helped nuance, rather than contradict, the dominant pattern of exclusion, and they were incorporated into the theme to reflect the complexity of participants’ responses.

One specific message that participants found limiting was the phrase “move more, sit less”, which is a simplified version of the frequently cited WHO guidelines for reducing sedentary behavior [2,27]. For one participant who relied on a power chair to participate in community life, this messaging was perceived as exclusionary: “very offensive to say that to someone like me, I find it offensive. Don’t you think I want to move?” (P4WCC). In contrast, when participants were presented with the scientific definition of sedentary behavior [26], and understood that energy expenditure could interrupt sedentary behavior, they responded more positively to the intention behind these recommendations. Some articulated a meaningful shift in perspective, recognizing that “you can be active in a seated position, but that, then, is, you know, explained by the definition of sedentary” (P6WLI). This prompted discussion around terminology suggesting that while less attention-grabbing, the scientific term “sedentary behavior” may be more appropriate and inclusive than villainizing “sitting” when addressing sedentary behavior in the context of SCI.

## 4. Discussion

Using reflexive thematic analysis, this study explored how individuals with SCI experience sedentary behavior and how they interpret media messages related to these behaviors. The following discussion will return the focus back to the research questions: (i) What do people with SCI know and believe about sedentary behavior and its effects on health and well-being? (ii) How do people with SCI experience prolonged periods of sitting still, and what are the contexts of these behaviors? (iii) What do people with SCI think about sedentary behavior messaging in the media?

### 4.1. What Do People with SCI Know About Sedentary Behavior?

People with SCI had a general awareness of sedentary behavior, often recognizing the term and its presence in public health messaging. However, their understanding was typically based on headlines and included a common misconception that increasing PA automatically offsets sedentary time [23,49]. Despite this conflation, participants still viewed sitting and sedentary behavior as detrimental to their health.

Lack of knowledge has been identified as a barrier to reducing sedentary behavior in older adults and working populations, yet studies vary widely in how they define and measure the depth of this knowledge [50,51]. As with other health behaviors (PA, smoking), basic awareness of risk associated with a behavior is rarely sufficient to drive meaningful behavior change [24,52]. Individuals with SCI need clearer guidance on the health risks of sedentary behavior (distinct from physical inactivity) and practical, achievable strategies for interruption that align with their capabilities. Knowledge with greater detail on how to make changes can increase healthy behaviors such as PA participation [25,53]. Future research should explore the detail of knowledge necessary to support meaningful reductions in sedentary behavior in people with SCI.

### 4.2. How Do People with SCI Experience Sedentary Behavior?

Individuals with SCI discussed sedentary behavior as shaped by their physical symptoms. Discomfort, fatigue, and concerns about secondary complications such as pressure sores shaped their experience. These symptoms often triggered negative emotions, including pain-related distress and anxiety about skin injury. Yet participants also sought balance, with some describing sedentary behavior as restorative and a normal part of daily life. This perspective reflects a duality also observed in older adults, where sedentary behavior is acknowledged for its negative health impact, but certain sedentary activities are appreciated for their restful and recuperative qualities [54,55]. Given the limited research on the lived experience of sedentary behavior among individuals with SCI, parallels can be drawn from studies of older adults, who constitute the largest group of wheelchair users and individuals with mobility disabilities [56,57,58].

Despite participants attributing physical symptoms to prolonged sitting, physiological evidence linking sedentary behavior to specific health outcomes in SCI remains limited [6]. The complexity and heterogeneity of SCI make it challenging to isolate symptoms that are directly influenced by sedentary time [59]. For example, the general population can expect increases in blood pressure and stiffening of arteries over a period of uninterrupted sitting [60,61]. People with SCI may not experience this phenomenon due to the denervation of the sympathetic nervous system below injury level. A survey study reported that prolonged sitting can exacerbate pain among people with SCI [62]. Although mechanistic evidence remains limited, participants’ interpretations of causality are meaningful within their lived experience. From a constructivist perspective, these attributions reflect the embodied knowledge through which individuals with SCI make sense of their symptoms, and they hold analytic value regardless of whether biomedical pathways have been empirically established. This underscores the broader need for foundational research that can accurately quantify sitting time among people with SCI before meaningful distinctions between symptom causes can be drawn.

### 4.3. What Do People with SCI Think About the Media Portrayal of Sedentary Behavior?

Participants in this study reacted strongly to alarmist headlines such as “sitting will kill you”, which they found particularly distressing. These headlines are examples of fear appeals [31]. Fear appeals can be effective for prompting single actions of health-related behaviors, especially disease detection behaviors such as cancer screening, when both perceived threat and perceived efficacy are high [63]. However, the effectiveness of fear appeals is mixed and highly context-dependent. For repeated prevention behaviors such as interrupting sedentary time, and particularly when individuals perceive low efficacy for making behavior changes, fear appeals tend to lose impact or become counterproductive [63]. Messages that imply everyone can simply stand or walk to prevent severe health consequences from sitting will provoke emotional and defensive reactions. Such messaging overlooks the lived realities of people with SCI and can feel exclusionary or dismissive, undermining its intended impact [27,64].

Participants appreciated the distinction between “sitting” and “sedentary behavior”. People with SCI noted that activities like wheelchair sports or propulsion involve “active sitting” and should not be equated with passive sitting such as sitting in one position for work or screen time. To leverage this distinction, public health messaging should highlight the difference between active and passive sitting [26]. Participants also called for SCI-specific resources that reflect their lived experience. Headlines which claimed “sitting is the new smoking” were shown to participants. In addition to participants taking offense to this claim, these comparisons between sitting and smoking are misleading and scientifically flawed, potentially undermining public understanding and tobacco control efforts [30].

### 4.4. Study Evaluation and Reflections

As the primary author, I acknowledge certain limitations in this study, many of which are common in qualitative interview research. Notably, I do not have lived experience with SCI, and my academic background in sedentary behavior has primarily focused on its physiological effects, rather than the psychological dimensions. As is consistent with rich qualitative methods, subjectivity and emotion within the process are celebrated as they cannot be fully removed, as is assumed in objective methods [65,66]. We acknowledged our unique viewpoints through maintaining a reflexive journal and reviewing the interview guide with multiple authors who implemented questions from multiple viewpoints. Additionally, because interviews were conducted via Zoom, consistent observation of non-verbal cues or environmental context was not possible, though this aligns with our focus on participants’ accounts rather than behavioral cues. As previously noted, directly asking participants about sedentary behavior may have inadvertently primed them to overemphasize the presence of sitting in their lives. Similarly, when exploring their reactions to fear-based messaging, the absence of balanced media representations may have biased their responses toward negative interpretations. These influences are inherent to qualitative inquiry in which participants are intentionally invited to reflect on specific phenomena, and in this case were necessary to elicit the perspectives required to address this study’s research questions. While these lines of questioning may have shaped participants’ focus on sedentary behavior and its portrayal in public messaging, they were essential for addressing this study’s core research questions.

While generalizability is not a common goal within a constructivist approach, this does not absolve qualitative researchers from considering how their findings may be meaningfully extended beyond the immediate study sample [67]. Instead, qualitative research allows for representational generalization or transferability. These concepts acknowledge the potential for findings to resonate with broader populations while still respecting the uniqueness of individual experiences [68]. In the present case, many of the themes identified may be relevant not only to this sample but also to people with SCI who do not ambulate, other people with disabilities, and wheelchair users. While these findings are not necessarily statistically representative, they may hold conceptual relevance for others in similar contexts.

## 5. Conclusions

This study is among the first to gather the knowledge and experiences of sedentary behavior from people with SCI, which includes their understanding, their lived experience, and how they receive messages in the media about the risks of sitting. The negative effects of sitting were known to people with SCI through their own experiences, peers, and identity as a person with SCI. While acknowledging the effects of sedentary behavior, people with SCI focused more often on increasing their PA rather than reducing sedentary behavior. Media including fear appeals were met with defense and a lack of engagement by people with SCI. In conclusion, sedentary behavior research among people with SCI remains a critical gap, and future evidence should be translated into sedentary behavior and sitting media that are meaningfully more inclusive.

## Figures and Tables

**Table 1:** Participant characteristics.

Gender		
Men	6	(50%)
Women	6	(50%)
Level of Injury		
Cervical	6	(50%)
Thoracic	4	(33%)
Lumbar	2	(17%)
Completeness of Injury		
Incomplete	8	(67%)
Complete	4	(33%)
Primary Mode of Community Mobility		
Manual Wheelchair	7	(58%)
Power Wheelchair	4	(33%)
A Mix of Wheelchairs	1	(8%)
Capable of Taking Standing Breaks		
No	5	(42%)
Yes, with a standing frame/aid	2	(17%)
Yes, without a standing frame/aid	5	(42%)
Age (years Mean ± SD)	49.2	12.7
Time Since Injury (years Mean ± SD)	17.3	8.6
Leisure Time Physical Activity (Total minutes per week, median days × median minutes)	(IQR: 25, 75)	
Mild Intensity Aerobic	45.0	7.5, 183.8
Moderate Intensity Aerobic	40.0	0.0, 183.8
Vigorous Intensity Aerobic	15.0	0.0, 101.3
Resistance	22.5	0.0, 130.0

## Data Availability

Due to the sensitive and personal nature of the qualitative interview data, the full transcripts cannot be publicly shared. Participants did not consent to the open distribution of their interview materials. De-identified excerpts relevant to the findings are included within the article. Additional information may be available from the corresponding author upon reasonable request and subject to ethical approval.

## Supplementary Materials

The following supporting information can be downloaded at https://www.mdpi.com/article/10.3390/healthcare14183068/s1: File S1: Interview guide; File S2: Interview resource.
