# Supplementary material for: The Meaning of Sedentary Behavior for People with Spinal Cord Injury: A Reflexive Thematic Analysis
Source: Healthcare (Basel). 2026 Sep 17;14(18):3068. doi: 10.3390/healthcare14183068 (PMC13606633; doi:10.3390/healthcare14183068)
Supplement: Supplementary file 1 [file healthcare-14-03068-s001.zip › File S1. Interview guide.pdf]

Sedentary Behaviour and People with Spinal Cord Injury; Perceptions and Meaning of Passive Sitting

Project Research Questions

RQ#1: What do people with Spinal Cord Injury know and believe about Sedentary Behaviour and its effects on health and well-being?

RQ#2: How do people with Spinal Cord Injury experience prolonged periods of sitting still, and what are the contexts of these behaviours?

INTERVIEW GUIDE

PREAMBLE: I want to thank you for your time and participation in our research. As discussed, I am interested in sedentary behaviour and passive sitting which we will discuss and explore further in the interview. Over the next period of time, please feel free to bring up any experiences or beliefs that you feel are relevant. If you would not like to continue, you can withdraw at any time. The length of the interview is roughly 30-60 minutes, and I have pre-planned questions I would like to discuss. I'd like you to know that at any time I may ask relevant, but impromptu questions at any time. Finally, be confident that any information that you share will remain confidential and be presented in aggregate form, meaning your results will be part of a group and not connected to your identity. Do you have any questions before we begin?

Italics represents a potential probing question.

INTRODUCTION

1. To begin, I just want to know a little bit about your life. Can you tell me a little bit about yourself?
    a. What does a typical week look like for you? What about the weekend? (*prompts for work, hobbies, interests)*
    b. How much time do you spend at home, and what is that like? *(prompts for family, chores, Activities of Daily Living, gardening)*
    c. Do you do regular physical activity? *(Prompt for frequency, intensity, time, and type, and possibly change over time)*

KNOWLEDGE

1. What do you know about sitting still and its influence on your health?
    a. (*Where have you heard about sitting still? Can you describe what the source was and what the message you received was?)*
    b. *If no, probe about specific media sources or HCP messages*

2. Do you recognize the term sedentary behaviour?
    a. *(If yes, what do you know about the term?)*
    b. *(Where have you heard the term?)*

KNOWLEDGE – DEFINITIONS

From above, the term **sedentary behaviour** has a specific scientific meaning. The scientific definition of Sedentary Behaviour is "any behaviour while awake and sitting, lying, or reclining with a low energy expenditure (less energy than it takes a person without a disability to wash dishes [1.8 METs] or hold a baby [1.5 METs])" Sitting can be further clarified into passive sitting, which means sitting without expending energy.

1. What do you think about that definition?
    a. Does that resonate with you/mean anything to you?
    b. Does the posture portion of the definition feel important as it may impact health (sitting, lying, reclining)?
        i. *(Probe about passive sitting v. active sitting)*
    c. Do you spend more time engaging in passive sitting or active sitting?
2. What do you feel is a "prolonged" amount of sitting still time for you?
    a. *(What does this look like for you? Possibly prompt them to show)*

CONTEXT

1. When you find yourself sitting still, what setting are you most often in? (home, work, etc.)
    a. *(What other activities or things are you doing when you are regularly sitting still?)*
2. Do you spend the most time sitting still for long periods of time in your wheelchair or in different chairs that you transfer to? *(Why do you sit in one chair over the other?)*
3. When sitting still for prolonged periods of time, are you most often by yourself or with other people?
    a. (How does this influence the amount of time you end up sitting still?)
    b. (Do you feel differently or notice your sitting time differently depending on the people who are around you?)

ATTITUDES

Scientific literature and media sources have discussed negative consequences of sitting. For example, as a recommendation to improve health, the World Health Organization currently recommends that both adults with and without physical disabilities "move more, sit less." Since we know people with SCI may engage in their movement and physical activity while sitting, we will specifically ask you in this interview about **passive sitting**

which to us means sitting while also not engaging in physical activity such as wheeling or arm-cranking ergometry/handcycling.

1. How do you feel about the recommendation of people to “move more, sit less?”
    a. *As noted above, this is both the recommendation for adults, as well as people with physical disabilites. Is this a good recommendation for adults without disabilities?*
    b. *Is it a good recommendation for people with physical disabilities, and you?*
2. When you have been sitting still for a long time, how do you feel most of the time?
3. Can you tell me about times when sitting still for a long time feels good or feels like a positive activity for you?
    a. *(What was the setting around this? Which chair were you in? Where were you? Who was around you? What other activities were you doing?)*
4. Can you tell me about times when sitting still for a long time feels bad or feels like a negative activity for you?
    a. *(What was the setting around this? Which chair were you in? Where were you? Who was around you? What other activities were you doing?)*
5. Numerous headlines have reported about these new scientific findings with flashy language. (Show the headlines paper) Headlines can include “sitting is the new smoking” and “sitting too much can kill you.” As a person with an SCI who sits regularly, how does reading these headlines make you feel?

> In adults, higher amounts of sedentary behaviour are associated with detrimental effects on the following health outcomes: all-cause mortality, cardiovascular disease mortality and cancer mortality and incidence of cardiovascular disease, type 2 diabetes and cancer.
> It is recommended that:
>
> - **Adults should limit the amount of time spent being sedentary. Replacing sedentary time with physical activity of any intensity (including light intensity) provides health benefits;**
> - **To help reduce the detrimental effects of high levels of sedentary behaviour on health, adults should aim to do more than the recommended levels of moderate-to-vigorous physical activity.**
>
> *Strong recommendation*

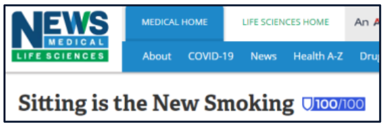

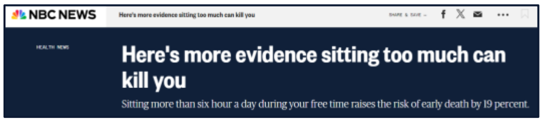

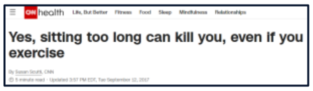

OPINION

Opinion: Sitting down all day is killing us. The cure is surprisingly simple — and difficult

THE CONVERSATION

Sitting all day is terrible for your health – here's a relatively easy way to counteract it

INTERRUPTIONS AND SIGNALS

1. What do you know about interrupting or breaking up long periods of sitting still?
    a. *What do you feel like interrupting periods of sitting still would do for you?*
2. Do you ever feel like you have been sitting still for too long?
    a. If you do, what sort of things tell you that you have been sitting for too long?
    b. What sort of things do you do when you feel you have been sitting too long?
3. If you can remember, did you have signals from when you were sitting too long before your injury?
    a. What told you that you had been sitting too long then?
    b. What did you do, or what activities did you do when you felt you were sitting too long?
4. Do you feel like you have control over your sitting time? Why or why not?
5. If you feel like you want to sit still for less time or break up periods of sitting for long periods of time, what kind of things do you do?
    a. If it was found that people with SCI should reduce total sitting time, what is a strategy do you think you would start with?

OVERARCHING THEMES AND CONCLUSIONS

1. How do you feel you experience sitting still and sedentary behaviour as a person with a spinal cord injury?
2. Do you feel you experience sitting still differently than your friends and family members who do not have a physical disability?
    a. *Why or why not? Elaborate?*
3. I have covered everything I had planned. Is there anything else you would like to add that we did not talk about today or go back to any topics? Or do you have comments to add at the end?

That concludes our interview. I would like to thank you for your time and input. This is incredibly valuable to us and we appreciate the time and emotional effort in discussing your experiences. Thank you.
