# Supplementary material for: The Meaning of Sedentary Behavior for People with Spinal Cord Injury: A Reflexive Thematic Analysis
Source: Healthcare (Basel). 2026 Sep 17;14(18):3068. doi: 10.3390/healthcare14183068 (PMC13606633; doi:10.3390/healthcare14183068)
Supplement: Supplementary file 1 [file healthcare-14-03068-s001.zip › File S2. Interview resource.pdf]

Sedentary Behaviour Definition

Any behaviour while awake and sitting, lying, or reclining with a low energy expenditure.

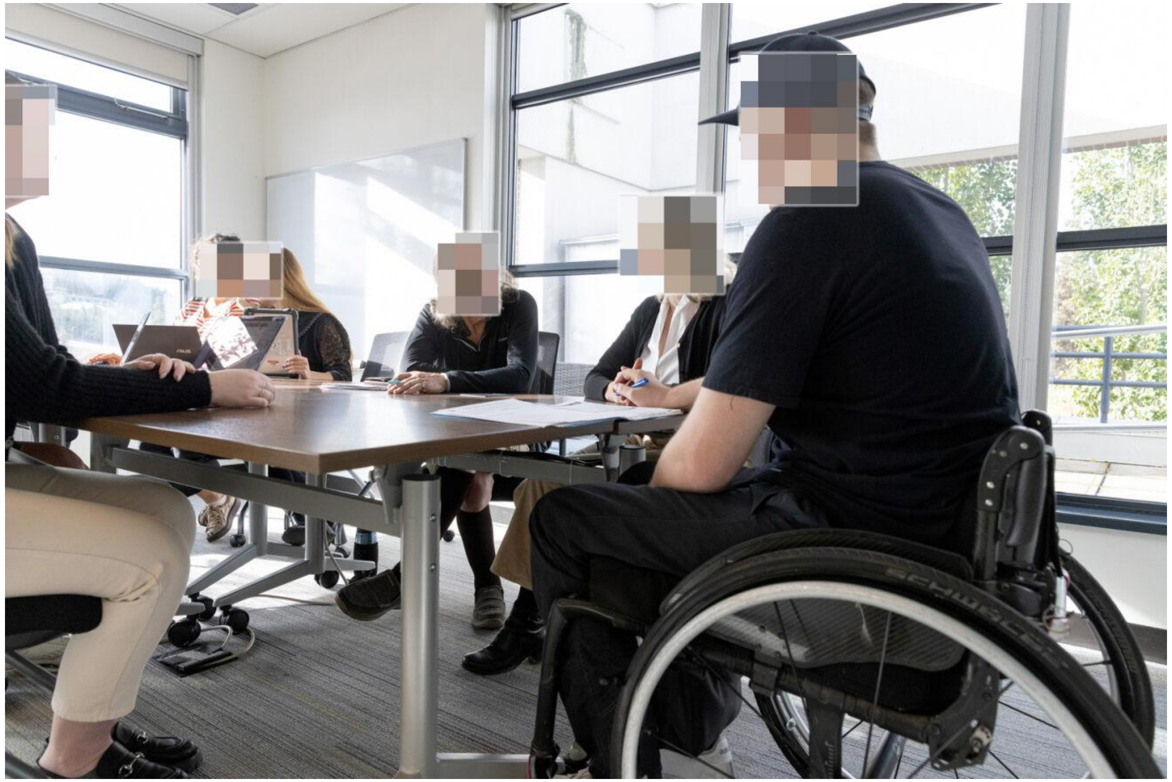

Individuals pixelated for publication

[26]

Activities of Daily Living Definition (ADLs)

The tasks of everyday life. Basic ADLs include eating, dressing, getting into or out of a bed or chair, taking a bath or shower, and using the toilet. Instrumental activities of daily living (IADL) are activities related to independent living and include preparing meals, managing money, shopping, doing housework, and using a telephone.

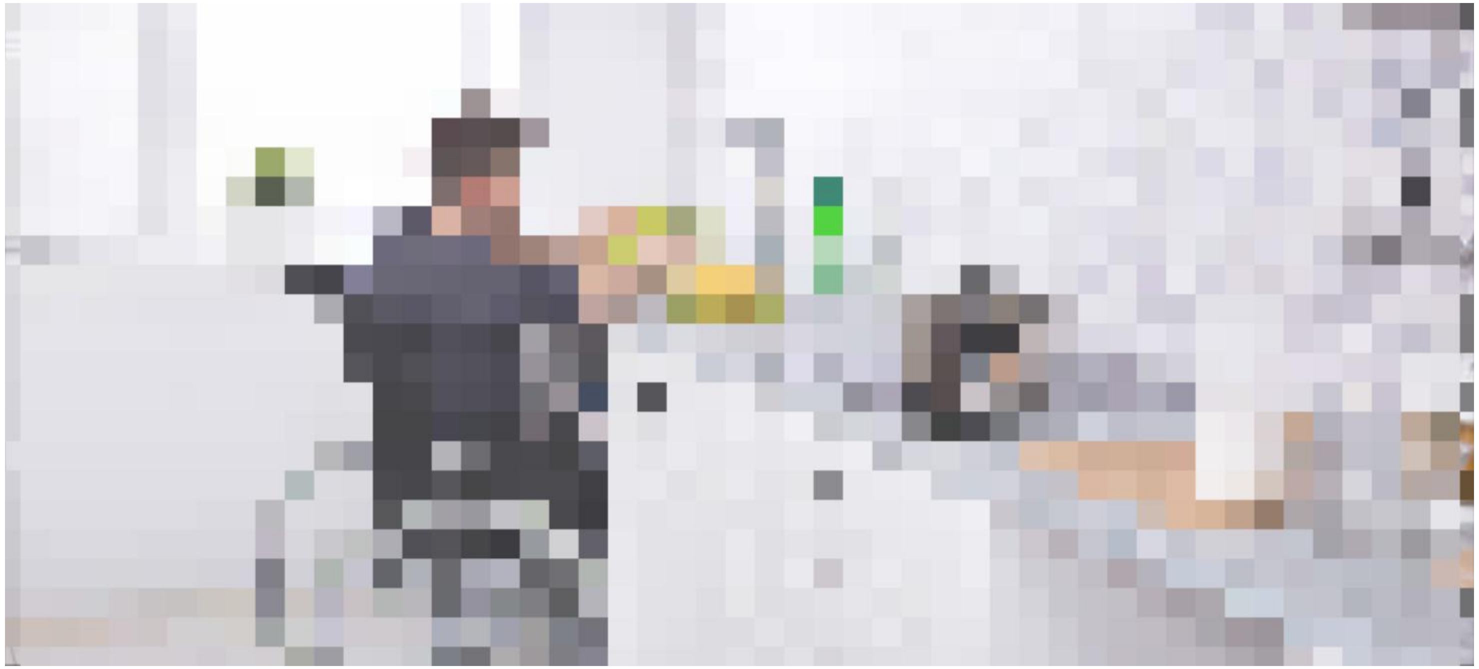

Image pixelated for publication

Person in a wheelchair doing dishes at an accessible sink

[46]

Passive Sitting v. Active Sitting

Passive sitting refers to any waking activity in a sitting posture characterized by an energy expenditure ≤1.5 METs.

Active sitting refers to any waking activity in a sitting posture characterized by an energy expenditure >1.5 METs

[26]

Tremblay, M. S., Aubert, S., Barnes, J. D., Saunders, T. J., Carson, V., Latimer-Cheung, A. E., Chastin, S. F. M., Altenburg, T. M., Chinapaw, M. J. M., & on behalf of SBRN Terminology Consensus Project Participants. (2017). Sedentary Behavior Research Network (SBRN) - Terminology Consensus Project process and outcome. *International Journal of Behavioral Nutrition and Physical Activity*, *14*(75). https://doi.org/10.1186/s12966-017-0525-8

Sedentary Behaviour Recommendation

In adults, higher amounts of sedentary behaviour are associated with detrimental effects on the following health outcomes: all-cause mortality, cardiovascular disease mortality and cancer mortality and incidence of cardiovascular disease, type 2 diabetes and cancer. It is recommended that:

- **Adults should limit the amount of time spent being sedentary. Replacing sedentary time with physical activity of any intensity (including light intensity) provides health benefits;**
- **To help reduce the detrimental effects of high levels of sedentary behaviour on health, adults should aim to do more than the recommended levels of moderate-to-vigorous physical activity.**

Strong recommendation

Bull, F. C., Al-Ansari, S. S., Biddle, S., Borodulin, K., Buman, M. P., Cardon, G., Carty, C., Chaput, J. P., Chastin, S., Chou, R., Dempsey, P. C., Dipietro, L., Ekelund, U., Firth, J., Friedenreich, C. M., Garcia, L., Gichu, M., Jago, R., Katzmarzyk, P. T., … Willumsen, J. F. (2020). World Health Organization 2020 guidelines on physical activity and sedentary behaviour. *British Journal of Sports Medicine*, *54*(24), 1451–1462. https://doi.org/10.1136/BJSPORTS-2020-102955

[40]

Sedentary Behaviour Media Headlines

NEWS MEDICAL LIFE SCIENCES | MEDICAL HOME | LIFE SCIENCES HOME | An A

About | COVID-19 | News | Health A-Z | Dru

Sitting is the New Smoking 100/100

Dutta, S. S. (2023, October 30). *Sitting is the new smoking*. News-Medical. https://www.news-medical.net/health/Sitting-is-the-New-Smoking.aspx

NBC NEWS | Here's more evidence sitting too much can kill you | SHARE & SAVE

HEALTH NEWS

Here's more evidence sitting too much can kill you

Sitting more than six hour a day during your free time raises the risk of early death by 19 percent.

Fox, M. (2018, June 29). *Here's more evidence sitting too much can kill you*. NBC News. https://www.nbcnews.com/health/health-news/here-s-more-evidence-sitting-too-much-can-kill-you-n887931

OPINION

Opinion: Sitting down all day is killing us. The cure is surprisingly simple — and difficult

Zomorodi, M., & Diaz, K. (2023, November 7). *Opinion: Sitting all day is terrible for your health — but here's what you can do about it*. *Los Angeles Times*. https://www.latimes.com/opinion/story/2023-11-07/sitting-sedentary-screen-device-work-health-diabetes-hypertension-exercise-fitness-npr

THE CONVERSATION

Sitting all day is terrible for your health – here's a relatively easy way to counteract it

Diaz, K. (2023, January 12). *Sitting all day is terrible for your health — here's a relatively easy way to counteract it*. The Conversation. https://theconversation.com/sitting-all-day-is-terrible-for-your-health-heres-a-relatively-easy-way-to-counteract-it-197507

CNN health | Life, But Better | Fitness | Food | Sleep | Mindfulness | Relationships

Yes, sitting too long can kill you, even if you exercise

By Susan Scutti, CNN

5 minute read · Updated 3:57 PM EDT, Tue September 12, 2017

Scutti, S. (2017, September 11). *Sitting increases risk of death, study says*. CNN. https://www.cnn.com/2017/09/11/health/sitting-increases-risk-of-death-study

[41-45]
